# Heterogeneity of prodromal Parkinson symptoms in siblings of Parkinson disease patients

**DOI:** 10.1038/s41531-021-00219-1

**Published:** 2021-09-07

**Authors:** Luca Baldelli, Sebastian Schade, Silvia Jesús, Sebastian R. Schreglmann, Luisa Sambati, Pilar Gómez-Garre, Claire Halsband, Giovanna Calandra-Buonaura, Astrid Daniela Adarmes-Gómez, Friederike Sixel-Döring, Corrado Zenesini, Chiara Pirazzini, Paolo Garagnani, Maria Giulia Bacalini, Kailash P. Bhatia, Pietro Cortelli, Brit Mollenhauer, Claudio Franceschi, PROPAG-AGEING consortium, Henry Houlden, Pietro Liò, Claudio Luchinat, Massimo Delledonne, Kevin Mills, Nancy L. Pedersen, Tiago Azevedo, Anna Bartoletti-Stella, Marta Bonilla-Toribio, Dolores Buiza-Rueda, Sabina Capellari, Mario Carriòn-Claro, Robert Clayton, Alessandra Dal Molin, Giovanna Maria Dimitri, Ivan Doykov, Cristina Giuliani, Sara Hägg, Jenny Hällqvist, Wendy Heywood, Ismael Huertas, Juulia Jylhävä, Miguel A. Labrador-Espinosa, Cristina Licari, Daniel Macias, Francesca Magrinelli, Juan Francisco Martín Rodríguez, Maria Giovanna Maturo, Giacomo Mengozzi, Gaia Meoni, Maddalena Milazzo, Christine Nardini, Nancy L. Pedersen, Maria Teresa Periñán-Tocino, Francesco Ravaioli, Claudia Sala, Simeon Spasov, Cristina Tejera-Parrado, Leonardo Tenori, Turano Paola, Dylan Williams, Luciano Xumerle, Elisa Zago, Marcella Broli, Dolores Buiza-Rueda, Patrizia De Massis, Rocio Escuela-Martin, Giovanni Fabbri, Anna Gabellini, Pietro Guaraldi, Henry Houlden, Stefania Macrì, Stefania Alessandra Nassetti, Cesa Lorella Maria Scaglione, Franco Valzania, Cilea Rosaria, Francesco Mignani, Rosario Vigo Ortega, Claudia Boninsegna, Silvia De Luca, Pablo Mir, Claudia Trenkwalder, Federica Provini

**Affiliations:** 1 Department of Biomedical and NeuroMotor Sciences (DiBiNeM), University of Bologna, Bologna, Italy; 2 Department of Clinical Neurophysiology, University Medical Center Göttingen, Göttingen, Germany; 3 Department of Neurosurgery, University Medical Center Göttingen, Göttingen, Germany; 4 Unidad de Trastornos del Movimiento, Servicio de Neurología y Neurofisiología Clínica, Instituto de Biomedicina de Sevilla, Hospital Universitario Virgen del Rocío/CSIC/Universidad de Sevilla, Seville, Spain; 5 Centro de Investigación Biomédica en Red sobre Enfermedades Neurodegenerativas (CIBERNED), Seville, Spain; 6 University College London (UCL), Institute of Neurology, London, United Kingdom; 7 IRCCS Istituto delle Scienze Neurologiche di Bologna, Bologna, Italy; 8 Paracelsus-Elena-Klinik Kassel, Kassel, Germany; 9 Neurologische Klinik, Philipps-University, Marburg, Germany; 10 Department of Experimental, Diagnostic and Specialty Medicine (DIMES), University of Bologna, Bologna, Italy; 11 Department of Neurology, University Medical Center Göttingen, Göttingen, Germany; 12 University of Cambridge, Cambridge, United Kingdom; 13 UCL Institute of Child Health Library, London, United Kingdom; 14 Personal Genomics Srl, Verona, Italy; 15 S. Maria della Scaletta Hospital, Imola, Italy; 16 Azienda Unità Sanitaria Locale di Bologna, Bologna, Italy; 17 University of Bologna, Bologna, Italy; 18 University of Oxford, Oxford, United Kingdom; 19 Karolinska Institutet, Stockholm, Sweden; 20 CERM, University of Florence, Florence, Italy; 21 Casa di cura Villa Baruzziana, Bologna, Italy; 22 University of Verona, Verona, Italy; 23 University of L’Aquila, L’Aquila, Italy; 24 Giotto Biotech srl, Florence, Italy; 25 Consiglio Nazionale delle Ricerche, Roma, Italia; 26 Azienda USL-IRCCS di Reggio Emilia, Reggio Emilia, Italy

**Keywords:** Risk factors, Parkinson's disease, Predictive markers

## Abstract

A prodromal phase of Parkinson’s disease (PD) may precede motor manifestations by decades. PD patients’ siblings are at higher risk for PD, but the prevalence and distribution of prodromal symptoms are unknown. The study objectives were (1) to assess motor and non-motor features estimating prodromal PD probability in PD siblings recruited within the European PROPAG-AGEING project; (2) to compare motor and non-motor symptoms to the well-established DeNoPa cohort. 340 PD siblings from three sites (Bologna, Seville, Kassel/Goettingen) underwent clinical and neurological evaluations of PD markers. The German part of the cohort was compared with German de novo PD patients (dnPDs) and healthy controls (CTRs) from DeNoPa. Fifteen (4.4%) siblings presented with subtle signs of motor impairment, with MDS-UPDRS-III scores not clinically different from CTRs. Symptoms of orthostatic hypotension were present in 47 siblings (13.8%), no different to CTRs (*p* = 0.072). No differences were found for olfaction and overall cognition; German-siblings performed worse than CTRs in visuospatial-executive and language tasks. 3/147 siblings had video-polysomnography-confirmed REM sleep behavior disorder (RBD), none was positive on the RBD Screening Questionnaire. 173/300 siblings had <1% probability of having prodromal PD; 100 between 1 and 10%, 26 siblings between 10 and 80%, one fulfilled the criteria for prodromal PD. According to the current analysis, we cannot confirm the increased risk of PD siblings for prodromal PD. Siblings showed a heterogeneous distribution of prodromal PD markers and probability. Additional parameters, including strong disease markers, should be investigated to verify if these results depend on validity and sensitivity of prodromal PD criteria, or if siblings’ risk is not elevated.

## Introduction

Parkinson’s disease (PD) is the second most frequent neurodegenerative disease with a prevalence of 50–300 per 100,000 for all ages, the incidence ranges from 10 to 30 per 100,000 person-years in western countries^1^. Both prevalence and incidence increase nearly exponentially with age and peak at 6% after the age of 80^2,3^. Risk factors for PD neurodegeneration lie in the various and complicated interplay between genetics and the environment, both of which contribute to the intrinsic heterogeneous nature of the disease^4^. The contribution of genetics to PD risk is currently estimated at around 30%, explained by a few monogenic^5^, but mainly polygenic mechanisms^6^. Age is the greatest non-familial risk factor^7^.

A prodromal phase of PD, characterized by a variety of non-motor symptoms (NMS), may precede the motor manifestation of the disease by years or even decades. When rapid eye movement sleep behavior disorder (RBD) occurs, the brainstem including subcoeruleus and reticularis magnocellularis nuclei and the limbic system are already affected. Olfactory dysfunction, cognitive impairment, autonomic dysfunction, pain, or fatigue may be already present at early stages^8–10^, and can precede PD motor onset^11–13^ in the so-called PD prodromal phase^14,15^. NMS are present in the elderly general population as well, but a combination of two or more NMS increases the risk of developing PD, especially if combined with subtle motor impairment^16–18^. Apart from idiopathic RBD, which has been widely studied as a specific marker for PD and alpha-synucleinopathies^14^, constipation and hyposmia make up the highest risk (up to 3.4 and 5.2-fold, respectively)^14,19–22^, though being non-specific and common in the general elderly population^23–25^. Although these markers are still not widely used in clinical practice and show a heterogeneous presentation in prodromal patients, the high conversion rate from the premotor or prodromal phase of the disease to PD provides a unique opportunity to directly observe disease development^26^. Applying the Movement Disorders Society criteria for prodromal PD, which obtained a 98.8% specificity in a 10-year longitudinal study^17^, prodromal PD prevalence has been estimated around 2.4% in the general elderly population^27^.

As mentioned above, family history is associated with an increased risk of PD development^28^. A metanalysis on epidemiological, referral, and registry-based studies showed a 2.9-fold increased risk in first-degree relatives of PD patients and a 4.4-fold increased risk of PD development in PD siblings^29–32^. Some studies have specifically evaluated prodromal markers in these individuals^9,33,34^. More recently, PD first-degree relatives (siblings) were specifically found more likely to suffer from anxiety, depression, and clinically diagnosed RBD than controls^33^. However, data are limited and the exact prevalence of prodromal PD signs and symptoms in PD siblings, and how they progress, are currently unknown.

The present work aims to comprehensively assess motor and NMS in PD siblings, to potentially define a high-risk group for developing PD for future interventions. We, therefore, considered a cross-sectional multi-center group of PD siblings (Sibs) recruited within the framework of the European PROPAG-AGEING (PPG) project. Within PPG, PD siblings, who have not been diagnosed with a neurodegenerative disease and do not fulfill the definition criteria of PD, are recruited in order to validate candidate clinical and biological (genetic, epigenetic, transcriptional, metabolic, lipid, and/or glycan) PD markers, as extensively described in Pirazzini et al.^35^.

We aimed to describe the presence, distribution, and combination of motor and NMS in PD siblings and estimate their prodromal PD probability, and to compare the presented motor and NMS to the well-established longitudinal DeNoPa cohort^36^.

## Results

### Demographic and general clinical data

A total of 340 siblings (*n* = 141; 41.5% males) were included in the study from the three recruiting centers, their mean age was 62.13 ± 10.72; SAS siblings (Spanish Sibs) were the youngest aged 57.77 ± 11.17 years (*p* < 0.001). Four Sibs had two siblings affected with PD. Alcohol and coffee consumption were more frequent among German Sibs (57.7% and 88.2%, respectively), while Spanish Sibs had the highest per capita alcohol daily dose (20.51 ± 20.92 g/day; *p* < 0.001). Regarding comorbidities, hypothyroidism was more prevalent among German Sibs (19.2%; *p* < 0.001).

In order to exclude national clusterization, we compared the 120 German siblings with 159 unrelated German de novo PD patients (dnPDs) and 109 controls (CTRs), who showed a uniform mean age (*p* = 0.875). In comparison to dnPDs and CTRs, more German Sibs were men (46% vs 38.6%; *p* < 0.001), had higher education, and were more active smokers (*p* < 0.001). DeNoPa PDs presented with a higher BMI than German Sibs (*p* = 0.001). CTRs presented with the highest prevalence of dyslipidemia and the lowest of hyperuricemia (CTRs vs German Sibs: *p* < 0.001 and 0.048, respectively).

Demographic data and comorbidities are shown in Table 1; consumptions and medications intake can be found in Supplementary Table 1, blood test data in Supplementary Table 2.

**Table 1:** Demographic data and comorbidities of Siblings and DeNoPa cohorts.

	PROPAG-AGEING Siblings’ cohort				DeNoPa cohort		Siblings’ comparisons		Comparisons with DeNoPa			
	Total Sibs (*n* = 340)	Italian Sibs (*n* = 100)	Spanish Sibs (*n* = 120)	German Sibs (*n* = 120)	dnPDs (*n* = 159)	CTRs (*n* = 109)	ISNB vs SAS vs UMG		dnPDs vs German Sibs		CTRs vs German Sibs	
							*p*	Adj. *p* (UMG vs ISNB; UMG vs SAS; ISNB vs SAS)	OR (CI)/*β*_coef._ (CI)	Adj. p	OR (CI)/*β*_coef._ (CI)	Adj. *p*
Men^a^	141 (41.5%)	44 (44.0%)	51 (42.5%)	46 (38.6%)	105 (66.0%)	66 (60.6%)	0.688	–	**3.13** (1.91–5.12)	**<0.001**	**2.47** (1.45–4.20)	**0.001**
Age (yrs.)^a^	62.13 ± 10.72	63.64 ± 9.64	57.77 ± 11.17	65.23 ± 9.73	65.25 ± 9.70	64.71 ± 6.85	**<0.001**	–	0.03 (−2.11–2.17)	0.981	−0.51 (−2.85–1.83)	0.669
Age difference with PD Sibs (yrs.)^a^	−1.09 ± 6.46	−3.24 ± 6.63	0.42 ± 6.53	−0.78 ± 5.79	–	–	**<0.001**	–	–	–	–	–
Age at PD onset in PD Sibs (yrs.)^a^	56.21 ± 10.71	58.14 ± 8.78	50.31 ± 10.17	60.32 ± 10.24	–	–	**<0.001**	–	–	–	–	–
Education (yrs.)^a^	11.68 ± 4.35	11.12 ± 4.09	10.47 ± 5.00	13.39 ± 3.21	9.93 ± 2.98	10.11 ± 2.97	**<0.001**	–	−**3.49** (−4.26–−2.73)	**<0.001**	−**3.16** (−3.99–−2.33)	**<0.001**
BMI (kg/m^2^)^a^	26.55 ± 4.28	26.24 ± 4.15	27.33 ± 4.19	26.03 ± 4.40	27.79 ± 4.48	26.69 ± 4.32	**0.045**	–	**1.76** (0.71–2.81)	**0.001**	0.65 (−0.50–1.80)	0.266
Comorbidities												
Hypertension	123 (36.3%)	29 (29.0%)	38 (31.7%)	56 (46.7%)	96 (60.4%)	53 (48.6%)	**0.012**	**0.004**; 0.165; 0.207	1.19 (0.66–2.16)	0.559	0.81 (0.43–1.52)	0.541
Diabetes	31 (9.3%)	4 (4.0%)	16 (13.3%)	11 (9.6%)	70 (10.7%)	8 (7.3%)	0.058	0.071; 0.091; **0.001**	0.98 (0.38–2.52)	0.968	0.73 (0.24–2.20)	0.579
Dyslipidemia	86 (25.5%)	22 (22.0%)	33 (27.5%)	31 (26.1%)	68 (42.8%)	64 (58.7%)	0.687	0.470; 0.705; 0.168	1.62 (0.89–2.94)	0.117	**3.00** (1.59–5.64)	**0.001**
Hyperuricemia	19 (5.6%)	4 (4.0%)	7 (5.7%)	8 (6.7%)	13 (8.2%)	4 (3.7%)	0.688	0.119; 0.317; 0.501	0.60 (0.19–1.89)	0.378	**0.23** (0.05–0.99)	**0.048**
Hypothyroidism	31 (9.1%)	4 (4.0%)	4 (3.3%)	23 (19.2%)	19 (11.9%)	10 (9.2%)	**<0.001**	**0.001**; **0.001**; 0.341	0.62 (0.26–1.47)	0.276	0.45 (0.18–1.18)	0.104
Cancer history	27 (7.9%)	7 (7.0%)	5 (4.2%)	15 (12.5%)	17 (10.7%)	9 (8.3%)	0.053	0.464; 0.331; 0.360	0.93 (0.38 −2.24)	0.867	0.66 (0.24–1.79)	0.412

Continuous variables are expressed in mean ± standard deviation, discrete in number (%). Adjustments have been made for age, sex, education, smoking, and coffee intake. Statistically significant coefficients and *p*-values are reported in bold.

*Total Sibs* total cohort of siblings of PD patients, *Italian Sibs* siblings coming from Azienda Unità Sanitaria Locale di Bologna—IRCCS Istituto delle Scienze Neurologiche di Bologna (Italy), *Spanish Sibs* siblings coming from Servicio Andaluz de Salud (Spain), *German Sibs* siblings coming from Universitätsmedizin Göttingen (Germany), *dnPDs* de novo PD patients from DeNoPa cohort, *CTRs* controls from DeNoPa cohort, *Adj. p* *p*-adjusted for covariates, *OR* odds ratio, *CI* 95% confidence interval, *β*_*coef.*_ coefficient of regression from linear regression, *PD Sibs* sibling affected with Parkinson’s Disease, *BMI* body mass index.

^a^These variables have not been adjusted.

### Motor evaluation and impact on daily living

Sibs obtained a median motor score of 0 (0–2) on the MDS-UPDRS part III. Fifteen (4.4%) Sibs presented with subtle signs of motor impairment (MDS-UPDRS part III score without action and postural tremor >6). German Sibs showed higher MDS-UPDRS III scores and reported a higher impact of motor symptoms on activities of daily living (MDS-UPDRS part II) than Italian and Spanish Sibs, even when adjusted for confounders.

Compared to CTRs, German Sibs presented with increased signs of motor impairment on the MDS-UPDRS part III (*p* < 0.001), 12 (10.0%) of them obtained a score greater than 6. German Sibs also presented with increased MDS-UPDRS part II scores than CTRs (*p* < 0.001).

Complete data on motor evaluation and motor impact on daily living are shown in Table 2.

**Table 2:** MDS-UPDRS in Siblings and DeNoPa cohorts.

	PROPAG-AGEING Siblings’ cohort				DeNoPa cohort		Siblings’ comparisons		Comparisons with DeNoPa			
	Total Sibs (*n* = 340)	Italian Sibs (*n* = 100)	Spanish Sibs (*n* = 120)	German Sibs (*n* = 120)	dnPDs (*n* = 159)	CTRs (*n* = 109)	ISNB vs SAS vs UMG		dnPDs vs German Sibs		CTRs vs German Sibs	
							*p*	Adjusted *p* (UMG vs ISNB; UMG vs SAS; ISNB vs SAS)	IRR (CI)	Adj. *p*	IRR (CI)	Adj. *p*
MDS-UPDRS												
Part I	2 (0–4)	1 (0–2)	2 (0–4)	3 (1–6)	5 (3–10)	2 (1–4)	**<0.001**	**<0.001**; **<0.001**; **<0.001**	**1.51** (1.32–1.71)	**<0.001**	**0.62** (0.53–0.73)	**<0.001**
Part II	0 (0–0)	0 (0–0)	0 (0–0)	0 (0–2)	8 (5–12)	0 (0–0)	**<0.001**	**<0.001**; **<0.001**; **<0.001**	**7.23** (5.97–8.76)	**<0.001**	**0.16** (0.10–0.26)	**<0.001**
Part III	0 (0–2)	1 (0–2)	0 (0–1)	1 (0–3.5)	22 (14–31)	0 (0–0)	**<0.001**	**<0.001**; **0.016**; 0.444	**10.54** (9.23–12.03)	**<0.001**	**0.27** (0.21–0.35)	**<0.001**
Part III without action and postural tremor	0 (0–2)	0 (0–2)	0 (0–0)	1 (0–3)	21 (13–31)	0 (0–0)	**<0.001**	**<0.001**; **<0.001**; 0.072	**10.98** (9.56–12.62)	**<0.001**	**0.19** (0.13–0.26)	**<0.001**
Part IV	0 (0–0)	0 (0–0)	0 (0–0)	0 (0–0)	0 (0–0)	0 (0–0)	0.331	–	–	–	–	–
Total	3 (1–7)	2.5 (1–4)	2 (0–7)	5.5 (3–11)	35 (24–50)	2 (1–5)	**<0.001**	**<0.001**;**<0.001**; **<0.001**	**5.07** (4.69–5.48)	**<0.001**	**0.45** (0.40–0.51)	**<0.001**

Subtle PD signs									OR (CI)	*p*	OR (CI)	*p*
Part II score > 1	43 (12.65%)	1 (1.0%)	11 (9.2%)	31 (25.8%)	154 (96.9%)	6 (5.5%)	**<0.001**	**<0.001**; **0.014**; **0.011**	**66.50** (22.20–199.20)	**<0.001**	**0.10** (0.03–0.33)	**<0.001**
Part III score > 1	119 (35.0%)	43 (43.0%)	23 (19.2%)	53 (44.2%)	159 (100%)	16 (14.7%)	**<0.001**	0.764; **0.029**; **0.017**	–	–	**0.29** (0.13–0.69)	**0.005**
Part III score > 6 without action and postural tremor	15 (4.4%)	1 (1.0%)	2 (1.6%)	12 (10.0%)	150 (94.3%)	0 (0.0%)	**0.016**	**0.009**; **0.032**; 0.192	**298.95** (69.71–1281.95)	**<0.001**	–	–

Continuous, not normally distributed variables are expressed in median (1st–3rd quartile), discrete in number (%). Adjustments have been made for age, sex, education, smoking, and coffee intake. Statistically significant coefficients and *p*-values are reported in bold.

Group acronyms are the same as explained in Table 1; *MDS-UPDRS* Movement Disorders Society: Unified Parkinson’s Disease Rating Scale, *Adj. p* *p*-adjusted for covariates, *IRR* incidence rate ratio, *OR* odds ratio, *CI* 95% confidence interval.

### Non-motor evaluation

Symptoms of orthostatic hypotension (OH) were the most frequent (47 Sibs; 13.8%), with a greater prevalence in German Sibs (*p* = 0.006) compared to Italian and Spanish Sibs, followed by constipation (40 Sibs; 11.8%). There was a tendency for more sexual dysfunction and orthostatic hypotension among German Sibs than CTRs (*p* = 0.058, *p* = 0.073, and *p* = 0.072, respectively). Complete autonomic data are presented in Table 3.

**Table 3:** Autonomic and olfaction data of siblings and DeNoPa cohorts.

	PROPAG-AGEING Siblings’ cohort				DeNoPa cohort		Siblings’ comparisons		Comparisons with DeNoPa			
	Total Sibs (*n* = 340)	Italian Sibs (*n* = 100)	Spanish Sibs (*n* = 120)	German Sibs (*n* = 120)	dnPDs (*n* = 159)	CTRs (*n* = 109)	ISNB vs SAS vs UMG		dnPDs vs German Sibs		CTRs vs German Sibs	
							*p*	Adj. *p* (UMG vs ISNB; UMG vs SAS; ISNB vs SAS)	OR (CI)/*β*_coef._ (CI)	Adj. *p*	OR (CI)/*β*_coef._ (CI)	Adj. *p*
*Autonomic function*												
Orthostatic hypotension symptoms	47 (13.8%)	5 (5.0%)	18 (15.0%)	24 (20.0%)	20 (12.9%)	9 (8.3%)	**0.005**	**0.006**; 0.194; 0.131	0.67 (0.31–1.47)	0.320	*0.43(0.17*–*1.08)*	*0.072*
Constipation symptoms	40 (11.8%)	10 (10.0%)	15 (12.5%)	15 (12.5%)	36 (22.6%)	5 (4.6%)	0.794	0.484; 0.845; 0.647	**2.63** (1.18–5.89)	**0.018**	0.42 (0.14–1.32)	0.139
Erectile/sexual	*n* = 151	*n* = 49	*n* = 60	*n* = 42	*n* = 155	*n* = 109						
Symptoms	17 (11.3%)	7 (14.3%)	5 (8.3%)	5 (11.9%)	35 (22.6%)	6 (5.5%)	0.612	0.411; 0.444; 0.832	1.40 (0.44–4.49)	0.571	*0.26* (0.06–1.05)	*0.058*
NMS Q26 f*s	0.36 ± 1.19	0.46 ± 1.27	0.17 ± 0.64	0.52 ± 1.16	0.80 ± 2.13	0.21 ± 1.26	0.546	0.352; 0.151; 0.630	−0.05 (−0.73–0.63)	0.887	*−0.64* (−1.35–0.06)	*0.073*
Sudomotor	*n* = 204	*n* = 100	*n* = 61	*n* = 43	*n* = 155	*n* = 109						
Symptoms	23 (11.3%)	7 (7.0%)	7 (11.5%)	9 (20.9%)	32 (20.6%)	16 (14.7%)	0.054	**0.012**; 0.117; 348	0.88 (0.34–2.30)	0.800	0.63 (0.23–1.76)	0.380
NMS Q30 f*s	0.44 ± 1.60	0.24 ± 1.30	0.40 ± 1.53	0.95 ± 2.10	1.15 ± 2.77	0.72 ± 2.18	**0.041**	**0.011**; **0.039**; 0.699	0.19 (−0.74–1.11)	0.691	−0.15 (−1.11–0.81)	0.758
*Olfaction*												
SSS score	9.51 ± 2.04	9.06 ± 2.35	9.72 ± 1.75	9.68 ± 2.00	5.78 ± 2.89	9.45 ± 1.86	0.090	0.060; 0.483; 0.149	**−3.67** (−4.32–−3.01)	**<0.001**	−0.08 (−0.78–0.62)	0.826
<10th percentile age and sex corrected^a^	22 (6.5%)	9 (9.0%)	8 (6.8%)	5 (4.2%)	74 (46.5%)	6 (5.5%)	0.347	0.236; 0.622; 0.392	**21.73** (8.02–58.91)	**<0.001**	1.43 (0.41–5.02)	0.577

Continuous variables are expressed in mean ± standard deviation, discrete in number (%). Adjustments have been made for age, sex, education, smoking, and coffee intake. Statistically significant coefficients and *p*-values are reported in bold, tendentially significant ones are in italics.

Group acronyms are the same as explained in Table 1; *Adj. p p*-adjusted for covariates, *OR* odds ratio, *CI* 95% confidence interval, *β*_*coef.*_ coefficient of regression from linear regression, NMS Q26 f*s frequency × severity score obtained on question no. 26 at the Non-Motor Symptoms Questionnaire, NMS Q30 f*s frequency × severity score obtained on question no. 26 at the Non-Motor Symptoms Questionnaire, *SSS* Sniffin’ Sticks Screening.

^a^Not statistically re-corrected for age and sex.

Among Sibs, the mean Sniffin’ Sticks Screening score was 9.51 ± 2.04; 22 Sibs (6.5%) were under the 10th percentile according to age and sex. Olfaction identification testing showed no difference between German Sibs and CTRs; dnPDs presented the worst performances (*p* < 0.001). Complete olfaction data are presented in Table 3.

Eight Sibs (2.4%) presented with anxiety disorder, 36 with depression (10.6%), there were no differences between the three recruiting centers. DnPDs suffered more from depression with a trend for a higher prevalence of anxiety disorders than German Sibs (*p* = 0.04 and 0.061, respectively); they also used more antidepressants (*p* = 0.001). Detailed results on affective-behavioral evaluation can be found in Table 4.

**Table 4:** Cognitive, affective-behavioral, and RBD data of siblings and DeNoPa cohorts.

	PROPAG-AGEING Siblings’ cohort				DeNoPa cohort		Siblings’ comparisons		Comparisons with DeNoPa			
	Total Sibs (*n* = 340)	Italian Sibs (*n* = 100)	Spanish Sibs (*n* = 120)	German Sibs (*n* = 120)	dnPDs (*n* = 159)	CTRs (*n* = 109)	ISNB vs SAS vs UMG		dnPDs vs German Sibs		CTRs vs German Sibs	
							*p*	Adj. p (UMG vs ISNB; UMG vs SAS; ISNB vs SAS)	OR (CI)/*β*_coef._ (CI)	Adj. p	OR (CI)/*β*_coef._ (CI)	Adj. *p*
*Cognition*		*n* = 119	*n* = 98									
MoCA adjusted	26.22 ± 3.36	25.39 ± 2.98	26.10 ± 3.81	27.04 ± 2.99	24.52 ± 3.09	25.95 ± 2.36	**<0.001**	**0.004**; **0.049**; 0.311	**−1.18(**−1.88–−0.47)	**0.001**	−0.10 (−0.84–0.64)	0.789
Visuospatial-executive	–	–	–	4.29 ± 1.02	4.18 ± 1.01	4.53 ± 0.69	–	–	0.05 (−0.20–0.31)	0.680	**0.42** (0.16–0.69)	**0.002**
Language	–	–	–	5.41 ± 0.77	5.12 ± 0.81	5.46 ± 0.80	–	–	0.10 (−0.12–0.33)	0.354	**0.43** (0.20–0.65)	**<0.001**
Attention, concentration, working memory	–	–	–	5.68 ± 0.74	5.45 ± 0.93	5.71 ± 0.54	–	–	−0.08 (−0.30–0.14)	0.489	0.19 (−0.03–0.42)	0.096
Abstraction	–	–	–	1.83 ± 1.96	1.23 ± 1.46	1.29 ± 1.56	–	–	**−0.37** (−0.53–−0.21)	**<0.001**	**−0.28** (−0.44–−0.12)	**0.001**
Short-term memory	–	–	–	3.35 ± 1.56	2.00 ± 1.67	2.48 ± 1.57	–	–	**−0.93** (−1.37–−0.49)	**<0.001**	**−0.50** (−0.95–−0.05)	**0.030**
Orientation	–	–	–	5.93 ± 0.30	5.94 ± 0.24	5.98 ± 0.14	–	–	0.037 (−0.034–0.109)	0.304	**0.076** (0.003–0.149)	**0.041**
Abnormal MoCA	109 (32.2%)	44 (44.0%)	36 (30.3%)	29 (24.2%)	70 (58.8%)	41 (41.8%)	**0.006**	**0.015**; 0.200; 0.203	**1.97** (1.04–3.70)	**0.036**	1.14 (0.58–2.24)	0.708
*Affective-behavioral*												
Anxiety disorder	8 (2.4%)	3 (3.0%)	2 (1.7%)	3 (2.5%)	13 (8.2%)	3 (2.8%)	0.803	0.427; 0.844; 0.228	*4.34* (0.94–20.1)	*0.061*	1.53 (0.25–9.50)	0.646
Depression	36 (10.6%)	7 (7.0%)	13 (10.8%)	16 (13.3%)	35 (22.0%)	17 (15.6%)	0.313	0.135; 0.392; 0.699	**2.30** (1.04–5.11)	**0.040**	1.39 (0.59–3.29)	0.457
*RBD*												
Clinical (RBDSQ) - Score	1.71 ± 1.85	1.94 ± 1.82	1.26 ± 1.78	1.98 ± 1.88	3.65 ± 2.81	2.38 ± 2.23	**<0.001**	0.425; **0.002**; **0.005**	**1.36** (0.70–2.02)	**<0.001**	0.32 (−0.36–1.00)	0.359
Positive	27 (8.0%)	9 (9.0%)	6 (5.0%)	12 (10.1%)	43 (28.7%)	17 (16.0%)	0.314	0.245; **0.030**; 0.213	**2.85** (1.24–6.57)	**0.014**	1.45 (0.58–3.61)	0.429
vPSG-confirmed	*n* = *147*	*n* = *98*	–	*n* = *49*	*n* = *159*	*n* = *109*	0.687	0.882	**8.93** (1.83–43.53)	**0.007**	0.55 (0.07–4.37)	0.576
	3 (2.0%)	1 (1.0%)		2 (4.3%)	40 (25.3%)	2 (1.8%)						

Continuous variables are expressed in mean ± standard deviation, discrete in number (%). Adjustments have been made for age, sex, education, smoking, and coffee intake. Statistically significant coefficients and *p*-values are reported in bold, tendentially significant ones are in italics

Group acronyms are the same as explained in Table 1; *Adj. p p*-adjusted for covariates, *OR* odds ratio, *CI* 95% confidence interval, *β*_*coef.*_ coefficient of regression from linear regression, *MoCA* Montreal Cognitive Assessment, *RBD* REM Sleep Behavior Disorder, *RBDSQ* REM Sleep Behavior Disorder Screening Questionnaire, *vPSG* video-polysomnography.

^a^Not statistically re-corrected for age and sex.

Sibs obtained an adjusted MoCA mean score of 26.22 ± 3.36; 109 Sibs (32.2%) showed abnormal results (score < 26 points). The prevalence of individuals with an abnormal MoCA score was partially comparable among the centers, although the test score was higher in German Sibs (27.04 ± 2.99) compared to ISNB- and Spanish Sibs. German Sibs did not score differently from CTRs. DnPDs were two-fold more cognitively impaired on the MoCA (*p* = 0.036) and presented with lower scores when compared to German Sibs. When cognitive domains were considered in detail, German Sibs scored worse than CTRs in visuospatial/executive [0.42 (CI 0.16–0.69) points, *p* = 0.002] and language [0.43 (CI 0.20–0.65) points, *p* < 0.001] tasks, without being different from dnPDs, and performed better than both groups in abstraction and short-term memory tasks. Specific data on cognition can be found in Table 4.

Sibs scored a median of 2 (0–4) points on the MDS-UPDRS part I and was significantly higher in German Sibs (*p* < 0.001). In addition, German Sibs showed a lower non-motor burden than dnPDs (*p* < 0.001), but higher than CTRs (*p* < 0.001). MDS-UPDRS part I data are shown in Table 2.

### Sleep evaluation

One-hundred and forty-seven Sibs (49 German Sibs and 98 Italian Sibs) underwent vPSG. Mean sleep efficiency (SE) was 74.47 ± 18.48% and significantly higher in Italian Sibs (*p* < 0.001). Italian Sibs also showed a higher proportion of N3 sleep (*p* < 0.001). Three (2.1%) Sibs (2 German Sibs, 1 ISNB-Sib) had vPSG-confirmed RBD. All three RBD positive Sibs were negative on the RBDSQ (score < 5). According to the RBDSQ, 27 (8.0%) Sibs were positive for probable (clinical) RBD (pRBD). Among the 27 pRBD, 18 Sibs were investigated with a vPSG, which excluded RBD in each of them.

vPSG-confirmed RBD was not more prevalent in German Sibs than in CTRs (4.3% vs 1.8%, respectively), whilst it was more frequent in dnPDs (25.3%).

RBD results are shown in Table 4, complete vPSG data can be found in Supplementary Table 3.

### Prodromal PD probability

**Fig. 1:**
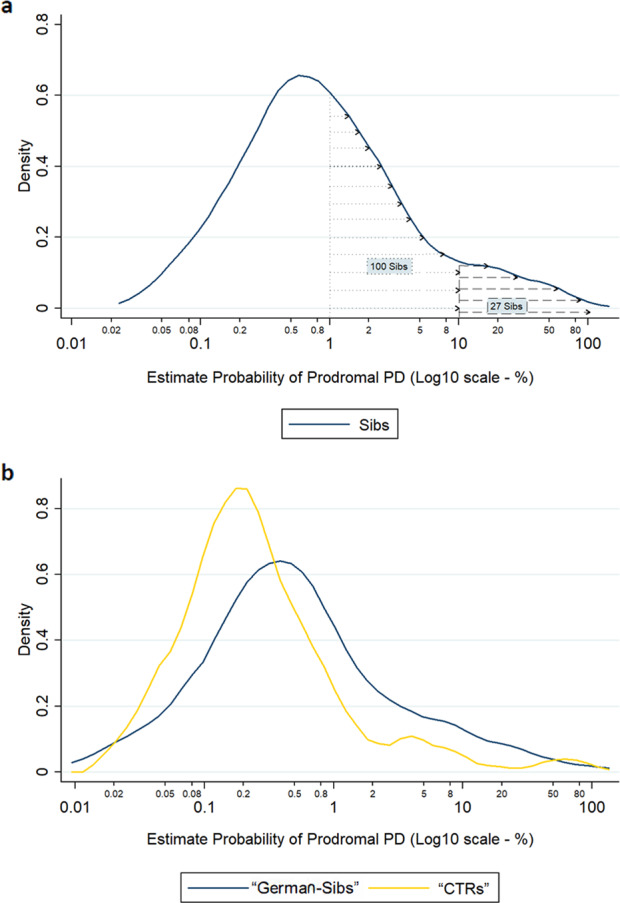
Prodromal PD probability of Siblings and DeNoPa cohorts. **a** Density distribution of prodromal PD probability. **b** Density distribution of prodromal PD probability in German Sibs and CTRs.

Prodromal PD probability was calculated according to MDS Research Criteria for Prodromal PD^15^, in their revised form^20^. The 300 Sibs over the age of 50 obtained a median prodromal PD probability of 0.75% (0.32–2.19%). One hundred and seventy-three Sibs had a <1% probability of having prodromal PD, 100 Sibs reached a probability between 1 and 10%, and 27 had a probability >10% (Fig. 1a). None of the 4 Sibs with two affected PD siblings had a probability greater than 10%. One sibling (0.33%) exceeded 80% probability (95.9%), fulfilling the criteria for Prodomal PD: he presented vPSG-confirmed RBD, constipation, and MDS-UPDRS III >6. The other two Sibs with confirmed RBD, the greatest prodromal PD risk factor, presented a probability of 40.2% and 36.8%, respectively.

One hundred and twelve German Sibs and 107 CTRs from DeNoPa over the age of 50 were evaluated for prodromal PD probability; family history of PD was not taken into consideration for this comparison. German Sibs presented with a median prodromal PD probability of 0.43% (0.17–1.09%), statistically greater than CTRs [0.215 (CI 0.12–0.45%), *p* < 0.001]. One UMG-Sib and no CTRs fulfilled the criteria for prodromal PD. The distribution of prodromal PD probability values was more heterogeneous in the German Sibs group compared to CTRs (Fig. 1b).

Detailed results on prodromal PD probability are shown in Table 5.

**Table 5:** Prodromal PD Probability and Markers in siblings and DeNoPa cohorts.

	PROPAG-AGEING Siblings’ cohort				DeNoPa cohort	Comparisons with DeNoPa	
	Total Sibs	Italian Sibs	Spanish Sibs	German Sibs	CTRs	CTRs vs German Sibs	
	*n* = 300	*n* = 92	*n* = 96	*n* = 112	*n* = 107	IRR (CI)/*β*_coef._ (CI)	Adj. *p*
*Prodromal PD calculation*							
Prodromal PD probability (%)	0.75 (0.32–2.19)	0.54 (0.27–1.27)	0.80 (0.28–2.32)	1.07 (0.43–2.69)	–	–	–
Log_10_(probability)	−0.04 ± 0.66	−0.20 ± 0.62	−0.06 ± 0.62	0.10 ± 0.70	–	–	–
Prodromal PD probability without fam. his. (%)	0.30 (0.13–0.91)	0.22 (0.11–0.57)	0.32 (0.11–0.94)	0.43 (0.17–1.09)	0.21 (0.12–0.45)	**0.64** (0.51–0.82)	**<0.001**
Log_10_(probability)	−0.40 ± 0.86	−0.49 ± 1.16	−0.45 ± 0.64	−0.28 ± 0.73	−0.58 ± 0.63	**−0.22** (−0.41–−0.03)	**0.025**
Prodromal PD positive	1 (0.33%)	0 (0.0%)	0 (0.0%)	1 (0.9%)	0 (0.0%)	–	–
Prodromal PD markers	*n* = 340	*n* = 100	*n* = 120	*n* = 120	*n* = 109	OR (CI)	*p*
# of markers	0 (0–1)	0 (0–1)	0 (0–1)	0 (0–1)	0 (0–1)	0.75 (0.39–1.43)	0.388
≥2 markers	42 (12.3%)	8 (8%)	14 (11.7%)	20 (16.7%)	11 (10.1%)	0.53 (0.18–1.55)	0.247

Continuous, not normally distributed variables and ordinal variables are expressed in median (1st–3rd quartile), continuous, normally distributed variables are expressed in mean ± standard deviation, discrete in number (%). Adjustments have been made for age, sex, education, smoking, and coffee intake. Statistically significant coefficients and *p*-values are reported in bold.

Group acronyms are the same as explained in Table 1; *Adj. p p*-adjusted for covariates, *IRR* incidence rate ratio, *CI* 95% confidence interval, *β*_*coef.*_ coefficient of regression from linear regression, *Prodromal PD probability without fam.* *his.* prodromal PD probability computed without considering the history of PD in first-degree relatives, *Log*_*10*_*(probability)* base 10 logarithm of prodromal PD probability.

### Distribution of prodromal PD markers

One hundred and forty-six out of 340 Sibs (42.9%) presented at least one prodromal PD marker, of whom 104 (30.6%) had only one marker, 29 (8.5%) had 2 markers and 13 (3.8%) had 3 markers. The most frequently associated markers were constipation and depression in 11 Sibs, followed by symptoms of OH-constipation and symptoms of OH-depression (7 Sibs each) (Supplementary Fig. 1, Part A).

Fifty-nine out of 120 (49.2%) German Sibs and 40 out of 109 (36.7%) CTRs presented at least one prodromal PD marker. The most frequently associated markers in German Sibs were constipation and depression (6 siblings) (Supplementary Fig. 1, Part B i), while in CTRs impaired olfaction and depression (3 controls) (Supplementary Fig. 1, Part B ii) were present. No difference was present between German Sibs and CTRs in the number of prodromal PD markers, when correcting for not equally distributed variables (adjusted p = 0.388).

Results on prodromal PD markers distribution are shown in Table 5.

## Discussion

To the best of our knowledge, this is the largest multi-national PD siblings’ study and the first one extensively evaluating motor and NMS and video-polysomnographically-validated RBD in this population^9,33,34^. In our study, we used a multi-national recruitment design with the inherent advantage of avoiding the gap of specific population characteristics and preventing the limitation of generalizability of the results. As part of our methodological approach, we analyzed both the MDS estimate of prodromal PD probability^15,20^ and considered the distribution of prodromal PD markers.

The main findings of our study are the following:

1. PD siblings showed subtle signs of motor and non-motor impairment, but when comparing German Sibs with CTRs, the differences were either not significant or did not fulfill criteria for clinical relevance, as outlined by Horvath^37,38^. From the motor point of view, the siblings obtained motor results comparable to non-converters, low-risk individuals^16^, and the general elderly population^39^, also the prevalence of siblings with mild parkinsonian signs was comparable to the general population^40^.
2. Regarding NMS, no relevant differences were found among Sibs from the three centers and between German Sibs and CTRs. German Sibs showed a significant increase of NMS impact on activities of daily living (MDS-UPDRS part I) with respect to CTRs, even if they differed by only 1 point. Our data agree with one out of the only three studies evaluating NMS in PD relatives, which did not find any difference with healthy controls^9^. The other two studies found an increased prevalence of anxiety and depression in PD relatives^33,34^. These incongruences could be explained by the different cohorts considered (first-degree relatives in general vs siblings) and by the different methodologies used to assess depression and anxiety (questionnaires and database records vs medical history). Indeed, our results show that reactive anxiety and depression, possibly related to the fear of developing PD, is not an issue in our population.
3. The prevalence of participants with an abnormal MoCA score was partially comparable among the centers. German Sibs did not perform differently from CTRs according to MoCA total score. However, German Sibs performed significantly worse than CTRs in visuospatial-executive functions and language, becoming similar to dnPDs in these subtests. Occurrence and characteristics of cognitive decline before PD onset are poorly understood^41^. To date, just three prospective population studies^18,42,43^ have demonstrated how lower scores in executive, visuospatial, and language functions could increase the risk of developing PD, while one study found lower cognitive performances to be connected with a higher probability of prodromal PD^44^. Only one study considered cognition in PD relatives, without evaluation of specific cognitive domains, finding no differences with controls^33^.
4. PD siblings did not show a higher prevalence of vPSG-validated RBD compared to the general population. Indeed, of the 147 Sibs evaluated by vPSG, only 3 Sibs had confirmed RBD. Using RBDSQ, Liu et al. previously reported an RBD prevalence in PD Sibs of 13.3% against 3.6% in controls^33^, which we were not able to confirm in our cohorts. However, in our cohort, we did not find concordance between vPSG and RBDSQ, and it is possible that in previous studies the use of RBDSQ overestimated the prevalence of RBD in PD sibs as the RBDSQ has not been validated in the general population and its specificity is generally low^45–47^.
5. Although the Sibs cohort is expected to be at higher risk^29^, only one Sib out 300 met the criteria for probable prodromal PD; this prevalence is not higher than the findings in a population cohort of otherwise healthy elderly individuals^27^. In our settings, the estimate of prodromal PD probability fails to identify PD siblings as a cohort at high risk as a whole and, therefore, the criteria of prodromal PD, which are mainly driven by the presence of RBD, do not show a higher risk of PD in our cohort. Whether this result depends on the validity or better on the sensitivity of the prodromal PD criteria or if the risk is actually not elevated in PD siblings cannot reliably be answered by the current data.
6. PD siblings showed a heterogeneous distribution of prodromal PD probability when using the MDS criteria for prodromal PD, as highlighted by the comparison between German Sibs and German CTRs. Twenty-seven of 300 Sibs had a probability of more than 10% of having prodromal PD and 42 of 340 Sibs presented at least two PD markers, hence, at-risk subjects were few but highly impacted^48^. In fact, by evaluating the distribution of score values and combined markers a subgroup of higher-risk individuals emerged, which disappears, if the specific marker of RBD is added and calculated.

Collectively, these results suggest on one hand that a reappraisal of currently available tools is needed, as they are only in part able to grasp the higher risk of PD siblings as reported by epidemiological data. Strong markers from additional clinical, biochemical (such as abnormal synuclein in the cerebrospinal fluid or in tissues), and molecular (e.g., polymorphisms in known at-risk loci) parameters should be investigated to increase their sensitiveness and help in disentangling heterogeneity as an intrinsic feature of PD neurodegeneration process. In parallel with PD subtypes, heterogeneity can reside also in the prodromal state and without broadening our search we could miss many prodromal PD patients. On the other hand, our results did show that prodromal symptoms are very uncommon in siblings, which could suggest that the low sibling risk is not a question of partial penetrance, but that siblings despite being more epidemiologically at risk of developing PD would not stall at a prodromal stage before some of them actually manifested the disease. Indeed, recent evidence showed borderline sensitivity when applying prodromal PD to the incident or longitudinal not enriched cohorts^49,50^.

Our study has several limitations. First, data of a de novo PD cohort were only available for the German center and we, therefore, decided to compare only German Sibs with de novo PD and controls, thus reducing numbers and statistical power. Second, genetic tests for known PD variants were not carried out at this stage. Third, apart from sleep with vPSG and odor identification impairment, other NMS were evaluated using patient reports and questionnaires, without instrumental or objective measurements, especially for strong prodromal NMS such as orthostatic hypotension. No interrater testing or training was performed prior to the study in the three sites, therefore interrater variabilities could account for the differences in ratings. The sleep studies are only partially comparable, as there are inherent differences between the compared lab-based vPSG in Germany (UMG) and the home-based vPSG (ISNB), although both centers are experienced in PSG RBD diagnosis, while no vPSGs data were included from SAS.

On the other hand, our study described motor and non-motor clinical and video-polysomnographic features in the largest PD sibling cohort up to date, using a multi-center approach, thus excluding the risk of clustered genetic and environmental risk factors related to the geographical context, especially in enclosed communities^30^. Furthermore, PD patients and siblings were not related but originated from different families. Indeed, the previous studies^9,33,34^ showed strong similarities between PD patients and siblings due to familial clustering, affecting not only PD-related markers but also possibly biasing features.

Our study indicates that siblings have a heterogeneous distribution of motor and non-motor signs. Additional clinical, biochemical, and molecular parameters not currently included in MDS criteria, and a focalized analysis of the more at-risk subjects, should pave the way of future analyses within the project. Finally, a prospective follow-up of an expanded collaborative international cohort can represent the right path to disentangle the complex interplay between genetic and environmental factors, characterizing the neurodegenerative and heterogeneous process^14^ of Parkinson’s Disease.

## Methods

### Design

**Fig. 2:**
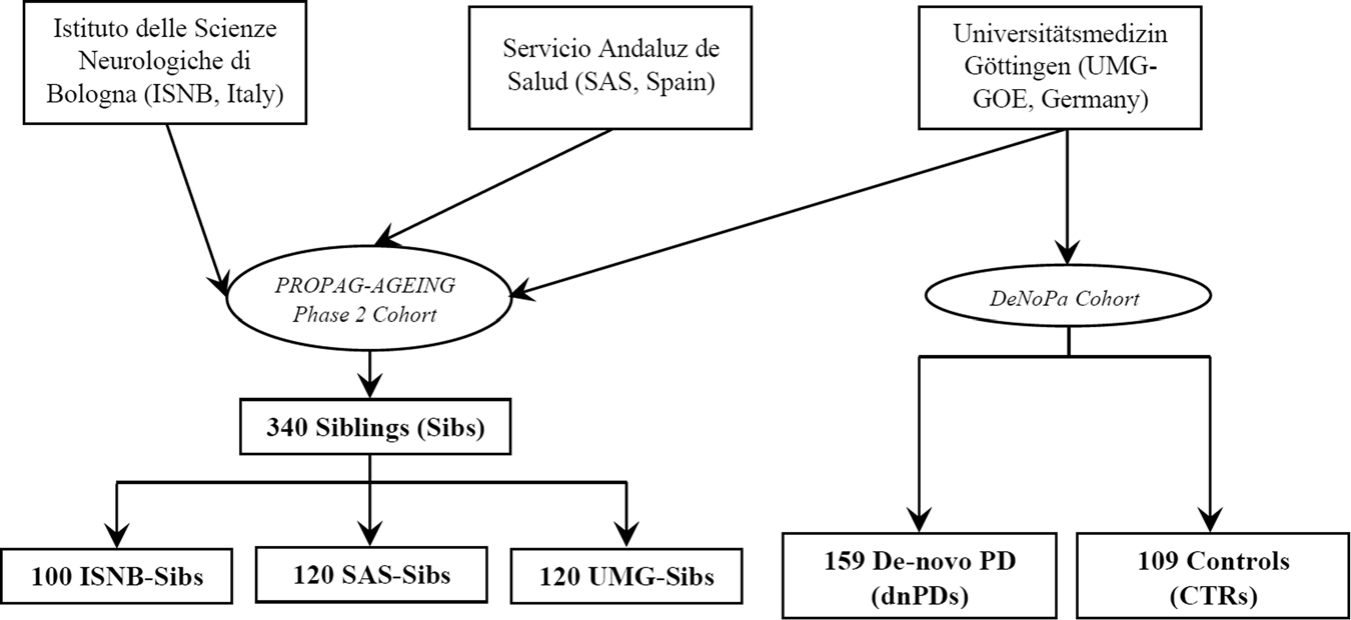
Study tree of the compared cohorts. Siblings were multicentrically recruited in Italy, Spain and Germany, German siblings were then compared with de novo PD patients and controls from the DeNoPa German cohort.

We report the description of the PPG siblings cohort (Sibs) multicentrically recruited over 29 months between September 2016 and January 2019 by three PPG partners: Azienda Unità Sanitaria Locale di Bologna—IRCCS Istituto delle Scienze Neurologiche di Bologna (ISNB, Italy), Servicio Andaluz de Salud (SAS, Spain) and Paracelsus-Elena Hospital, Kassel as part of Universitätsmedizin Göttingen (UMG-GOE, Germany). Sibs from Bologna and Kassel underwent video-polysomnography. To account for population-specific effects we subsequently compared sibling data from UMG-GOE (German-Sibs) with the baseline data of de novo PD patients (dnPDs) and controls (CTRs) from the DeNoPa cohort from the same center^36^ (Fig. 2).

### Study participants

Siblings of patients with a diagnosis of idiopathic PD according to the International Diagnostic Criteria (UK Brain Bank Criteria^51^), were recruited. The diagnoses of the affected PD patients were verified in each recruiting center by neurologists experienced in movement disorders, all belonging to the PROPAG-AGEING Consortium. Participants had to be over 18 years of age and could not have any active known/treated condition of the central nervous system (e.g., Alzheimer’s disease, vascular encephalopathies, multiple sclerosis) including PD; data on dnPDs and CTRs were retrieved from DeNoPa database^36^. DeNoPa is a large single-center cohort of early PD patients recruited at Paracelsus-Elena Hospital, Kassel including frequency-matched healthy controls. DeNoPa’s subjects evaluation includes motor signs, NMS, and a combination of diagnostic tests including olfactory testing, transcranial sonography of substantia nigra, and polysomnography (PSG)^36^.

### Investigations

Each recruited sibling received a full clinical evaluation including complete medical history, accounting for both comorbidities and medication, and a neurological examination performed by a neurologist experienced in movement disorder assessment. Motor symptoms were quantitatively assessed using the Unified Parkinson’s Disease Rating Scale (MDS-UPDRS) part III, while the impact on activities of daily living and NMS was assessed by the MDS-UPDRS parts II and I^52^. NMS were evaluated by means of validated questionnaires, such as the Non-Motor Symptoms Questionnaire (NMSQ—questions 26 and 30)^53^, the Rome III Diagnostic Criteria for Functional Constipation^54,55^, the Montreal Cognitive Assessment (MoCA)^56^ and the REM sleep behavior disorder screening questionnaire (RBDSQ)^46^. We also objectively assessed olfactory function using Sniffin’ Sticks Screening^57^, obtained selected laboratory and blood parameters (ISNB and UMG-GOE only), and carried out a whole night video-polysomnography (vPSG) in ISNB (at home) and UMG-GOE (in the sleep laboratory) recruited siblings. ISNB home-based vPSG was performed by means of Xltek Trex HD Video Ambulatory system and included three monopolar electroencephalogram channels (as recommended by the American Academy of Sleep Medicine’s Manual for the Scoring of Sleep and Associated Events^58^), electrocardiogram (EKG), electrooculogram (EOG), chin and limbs EMG (bilateral extensor carpi and tibialis anterior muscles), and toraco-abdominal respirogram. UMG-GOE lab-based vPSG was performed applying cardiorespiratory PSG and including bilateral monopolar central EEG with 2 channels, EOG, chin and bilateral tibialis anterior surface EMG, airflow registration, tracheal sound registration by microphone, thoracic and abdominal belts to measure respiratory movements, EKG, and oximetry. All patients were documented with an infrared video recording synchronized to the PSG. Further methodological details are described as published elsewhere^59^.

### Computation of prodromal PD probability and evaluation of prodromal PD markers

Prodromal PD probability for Sibs over the age of 50 was computed according to the revised MDS Research Criteria^20^, which also outlined the prodromal PD markers considered for evaluation. Life risk and prodromal markers were evaluated and accounted for each participant, independently of their age. Subsequent prodromal PD probability calculation taking into account prior probability was computed for those over the age of 50 (prior probability estimates are not available in the literature for younger individuals). Risk scoring was computed as defined in refs. ^15,20^.

Risk markers were evaluated as follows:

- Sex: biological sex was considered for the purpose. No cases of hermaphroditism or pseudohermaphroditism were present in our cohorts.
- Pesticide and solvent exposure: data not systematically available (NsA) in our cohort and therefore not computed.
- Non-use of caffeine: evaluated as defined in refs. ^15,20^.
- Smoking: evaluated as defined in refs. ^15,20^.
- First-degree relative with PD: this risk marker, positive for every sibling in our cohort, was evaluated in the first computation of prodromal PD probability, while it was not taken into account in the comparison between German siblings and healthy controls, to exclude obvious biasing in the calculation. Known gene mutation and polygenic risk scores were not taken into account.
- Substantia nigra hyperechogenicity: NsA data.
- Diabetes mellitus type II: retrieved from accurate medical history.
- Physical inactivity: NsA data.
- Low plasma urate in men: evaluated as defined in ref. ^20^.

Prodromal markers were evaluated as follows:

- REM sleep behavior disorder (RBD): evaluated as defined in refs. ^15,20^. However, when the prodromal markers were evaluated for their distribution rather than for the calculation of prodromal PD probability, only video-polysomnographically validated RBD was considered as positive RBD, to guarantee a homogeneous methodological approach.
- Nuclear medicine dopaminergic imaging: NsA data.
- Subthreshold parkinsonism: MDS-UPDRS-III >6 excluding postural and action tremor evaluation was considered.
- Olfactory loss: available sex and age correction, provided by the manufacturer, for olfactory identification testing was adopted. In order to maintain a conservative approach (to prevent overestimation of prodromal markers), only individuals under the 10th percentile according to age and sex were considered as having an objective olfactory dysfunction.
- Constipation: individuals fulfilling Rome III Diagnostic Criteria for Functional Constipation (thus excluding primarily gastrointestinal causes) were considered positive for this marker^54,55^.
- Excessive daytime somnolence: individuals with a score ≥3 on MDS-UPDRS question 1.8, thus responding positively to the following statement: “I sometimes fall asleep when I should not. For example, while eating or talking with other people.”
- Neurogenic orthostatic hypotension (OH): NsA data.
- Symptomatic OH: symptoms of OH were evaluated using a semi-structured clinical interview based on either Non-Motor Symptoms Questionnaire or MDS-UPDRS question 1.12.
- Erectile dysfunction in men: erectile dysfunction in men was evaluated using a semi-structured clinical interview based on Non-Motor Symptoms Questionnaire.
- Urinary dysfunction: individuals with a score ≥3 at MDS-UPDRS question 1.10, thus responding positively to the following statement: “Urine (control) problems cause a lot of difficulties with my daily activities, including urine accidents.”
- Depression (±anxiety): individuals were considered positive for depression (and/or anxiety) only when actual disorders, requiring pharmacological treatment and/or psychological assistance, were reported during the clinical interview.
- Global cognitive deficit: cognition was evaluated using the Montreal Cognitive Assessment (MoCA) in our cohort, which presented several issues of cutoff identification when individuals from different nationalities needed to be compared. Therefore, in order to maintain a conservative approach, MoCA score distribution was computed for each subgroup and nationality, individuals below 2 standard deviations were selected as cognitively impaired for prodromal marker selection. It should be noted that our approach is more conservative than the original methodology used by Heinzel et al. to compute the Global cognitive deficit risk score of 1.8, which was derived from studies using a cutoff of only 1 standard deviation^20^.

### Standard protocol approvals, registrations, and patient consents

We conducted the study according to the Declaration of Helsinki and all participants provided informed written consent. The study was approved by the local ethics committees of each recruiting PPG partner (UMG-GOE ethics committee no. of approval 19/5/16 of August 2016, ISNB ethics committee no. of approval 16018 of May 2016, SAS ethical committee no. of approval 2014/PI173 of September 2016).

### Database and statistical analysis

A designated online database was created for the study and collected demographic and laboratory data, clinically relevant comorbidities, and questionnaire results. All continuous normally distributed data were expressed as means and standard deviations (SD), while not normally distributed data were expressed as medians and interquartile ranges (IQR); the categorical data were expressed as absolute frequency and percentages (%).

Chi-squared, one-way analysis of variance (ANOVA) or Kruskal-Wallis test were used to investigate the differences between groups (German Sibs and DeNoPa’s dnPDs and CTRs) and PD siblings grouped by enrollment center (UMG-GOE, ISNB, SAS) and the demographic and lifestyle variables, comorbidities, medications, MDS-UPDRS parameters, NMS, blood tests, and macrostructure sleep data.

Multivariate logistic regression models, multivariate linear regression models, and multivariate Poisson regression models were used to evaluate the differences between groups and variables described above, adjusted for the following confounding variables of age, sex, years of education, smoking (cigarettes per day), and coffee intake (cups per day). The results were presented as odds ratios (OR) (95% confidence interval—CI) or β coefficient (95% CI) or incidence rate ratio (IRR) (95% CI).

Statistical analysis was performed using SPSS Statistics version 21 (IBM, Armonk, NY, USA) and Stata SE version 14.2 (StataCorp LLC, Texas, USA).

### Reporting summary

Further information on research design is available in the Nature Research Reporting Summary linked to this article.

## Supplementary information

Supplementary material

REPORTING SUMMARY

## Data Availability

Anonymized data and metadata will be shared by request from any qualified investigator.
