# Supplementary material for: Heterogeneity of prodromal Parkinson symptoms in siblings of Parkinson disease patients
Source: NPJ Parkinsons Dis. 2021 Sep 7;7:78. doi: 10.1038/s41531-021-00219-1 (PMC8423761; doi:10.1038/s41531-021-00219-1)
Supplement: Supplementary file 1 — Supplementary material [file 41531_2021_219_MOESM1_ESM.docx]

**Supplementary Note 1** - PROPAG-AGEING consortium

**Project Coordinator**

Claudio Franceschi, MD^7^

**Local Coordinators**

Kailash P. Bhatia, FRCP, MD,^6^ Henry Houlden, MD, PhD,^6^ Pietro Liò, PhD,^12^ Claudio Luchinat, PhD,^20^ Massimo Delledonne, MD,^14,22^ Kevin Mills, PhD,^13^ Nancy L. Pedersen, PhD,^19^ Paolo Garagnani, MD, PhD,^10^ Pablo Mir, MD, PhD,^4,5^ Claudia Trenkwalder, MD,^3,8^ Federica Provini, MD, PhD^1,7^

**Site Investigators**

Astrid Daniela Adarmes-Gómez, MD,^4,5^ Tiago Azevedo, PhDst,^12^ Maria Giulia Bacalini, PhD,^7^ Luca Baldelli, MD,^1^ Anna Bartoletti-Stella, PhD, ^7^ Marta Bonilla-Toribio, MSc, ^4,5^ Dolores Buiza-Rueda, Msc, ^4,5^ Giovanna Calandra-Buonaura, MD, PhD,^1,7^ Sabina Capellari, MD, PhD,^1,7^ Mario Carriòn-Claro, PhD,^4,5^ Robert Clayton, PhD,^13^ Pietro Cortelli, MD,^1,7^ Alessandra Dal Molin, PhD, ^14^ Giovanna Maria Dimitri, PhD, ^12^ Ivan Doykov, PhDst,^13^ Giuliani Cristina, PhD,^17,18^ Sara Hägg, PhD,^19^ Jenny Hällqvist, PhDst,^13^ Wendy Heywood, PhD,^13^ Ismael Huertas, PhD,^4,5^ Juulia Jylhävä, PhD,^19^ Miguel A. Labrador-Espinosa, MSc,^4,5^ Pilar Gómez-Garre, PhD,^4,5^ Silvia Jesús, MD, PhD,^4,5^ Cristina Licari, PhDst,^20^ Pietro Liò, PhD,^12^ Claudio Luchinat, PhD,^20^ Daniel Macias, MD,^4,5^ Francesca Magrinelli, PhD,^6,22^ Juan Francisco Martín Rodríguez, PhD,^4,5^ Massimo Delledonne, MD,^14,22^ Maria Giovanna Maturo, PhD,^23^ Giacomo Mengozzi, MSc,^7^ Gaia Meoni, PhD,^24^ Maddalena Milazzo, PhD,^10^ Kevin Mills, PhD,^13^ Brit Mollenhauer, MD,^8,11^ Christine Nardini, PhD,^25^ Nancy L. Pedersen, PhD,^19^, Maria Teresa Periñán-Tocino, MSc,^4,5^ Chiara Pirazzini, PhD,^7^ Francesco Ravaioli, PhD,^10^ Claudia Sala, PhD,^10^ Luisa Sambati, MD, PhD,^1,7^ Sebastian Schade, MD,^2,3^ Sebastian R. Schreglmann, MD, PhD,^6^ Simeon Spasov, PhDst,^12^ Friederike Sixel-Döring, MD,^8,9^ Cristina Tejera-Parrado, MSc,^4,5^ Leonardo Tenori, PhD,^20^ Turano Paola, PhD,^20^ Dylan Williams, PhD,^19^ Luciano Xumerle, PhD,^14^ Elisa Zago, MSc^14^

**Recruiting Investigators**

Astrid Daniela Adarmes-Gómez, MD,^4,5^ Luca Baldelli, MD,^1^ Marta Bonilla-Toribio, MSc, ^4,5^ Marcella Broli, MD, PhD,^7^ Dolores Buiza-Rueda, Msc, ^4,5^ Giovanna Calandra-Buonaura, MD, PhD,^1,7^ Sabina Capellari, MD, PhD,^1,7^ Mario Carriòn-Claro, PhD,^4,5^ Pietro Cortelli, MD,^1,7^ Patrizia De Massis, MD,^15^ Rocio Escuela-Martin,^4,5^ Giovanni Fabbri, MD,^16^ Anna Gabellini, MD,^7^ Pilar Gómez-Garre, PhD,^4,5^ Pietro Guaraldi, MD, PhD,^7^ Henry Houlden, MD, PhD,^6^ Silvia Jesús, MD, PhD,^4,5^ Stefania Macrì, MD,^21^ Brit Mollenhauer, MD,^8,11^ Stefania Alessandra Nassetti, MD, PhD,^7^ Luisa Sambati, MD, PhD,^1,7^ Sebastian Schade, MD,^2,3^ Sebastian R. Schreglmann, MD, PhD,^6^ Cesa Lorella Maria Scaglione, MD, PhD,^7^ Franco Valzania, MD, PhD^26^

**Technicians**

Cilea Rosaria, MTech,^7^ Francesco Mignani, MTech,^7^ Rosario Vigo Ortega, MTech^4,5^

**Study Nurses**

Claudia Boninsegna,^7^ Silvia De Luca ^7^

1. Department of Biomedical and NeuroMotor Sciences (DiBiNeM), University of Bologna, Bologna, Italy.
2. Department of Clinical Neurophysiology, University Medical Center Göttingen, Göttingen, Germany.
3. Department of Neurosurgery, University Medical Center Göttingen, Göttingen, Germany
4. Unidad de Trastornos del Movimiento, Servicio de Neurología y Neurofisiología Clínica, Instituto de Biomedicina de Sevilla, Hospital Universitario Virgen del Rocío/CSIC/Universidad de Sevilla, Seville, Spain.
5. Centro de Investigación Biomédica en Red sobre Enfermedades Neurodegenerativas (CIBERNED), Spain.
6. University College London (UCL), Institute of Neurology, London, United Kingdom
7. IRCCS Istituto delle Scienze Neurologiche di Bologna, Bologna, Italy
8. Paracelsus-Elena-Klinik Kassel, Kassel, Germany
9. Neurologische Klinik, Philipps-University, Marburg, Germany
10. Department of Experimental, Diagnostic and Specialty Medicine (DIMES), University of Bologna, Bologna, Italy.
11. Department of Neurology, University Medical Center Göttingen, Göttingen, Germany
12. University of Cambridge, Cambridge, United Kingdom
13. UCL Institute of Child Health Library, London, United Kingdom
14. Personal Genomics Srl, Verona, Italy
15. S. Maria della Scaletta Hospital, Imola, Italy
16. Azienda Unità Sanitaria Locale di Bologna, Bologna, Italy
17. University of Bologna, Bologna, Italy
18. University of Oxford, Oxford, United Kingdom
19. Karolinska Institutet, Stockholm, Sweden
20. CERM, University of Florence, Florence, Italy
21. Casa di cura Villa Baruzziana, Bologna, Italy
22. University of Verona, Verona, Italy
23. University of L'Aquila, L'Aquila, Italy
24. Giotto Biotech srl, Florence, Italy,
25. Consiglio Nazionale delle Ricerche, Roma, Italia
26. Azienda USL-IRCCS di Reggio Emilia, Reggio Emilia, Italy

**Supplementary Table 1.** Consumptions and medications intake data of siblings and DeNoPa cohorts.

|  | **PROPAG-AGEING Siblings’ Cohort** | | | | **DeNoPa Cohort** | | **Siblings’ Comparisons** | | **Comparisons with DeNoPa** | | | |
| --- | --- | --- | --- | --- | --- | --- | --- | --- | --- | --- | --- | --- |
|  | **Total Sibs**  ***(n=340)*** | **Italian Sibs *(n=100)*** | **Spanish Sibs *(n=120)*** | **German Sibs *(n=120)*** | **dnPDs *(n=159)*** | **CTRs**  ***(n=109)*** |  |  | **dnPDs vs.  German Sibs** | | **CTRs vs.  German Sibs** | |
|  |  |  |  |  |  |  | **p** | **Adj. p**  *(UMG vs ISNB; UMG vs SAS; ISNB vs SAS)* | **OR (CI) /  βcoef. (CI)** | **Adj. p** | **OR (CI) /  βcoef. (CI)** | **Adj. p** |
| **Consumption*** |  | | | | | | | | | | | |
| Smoke - Yes | 71 (20.9%) | 24 (24.0%) | 25 (20.8%) | 22 (18.3%) | 11 (6.9%) | 4 (3.7%) | 0.565 |  | **0.42**  (1.19 – 0.92) | **0.030** | **0.26**  (0.08 – 0.79) | **0.018** |
| - Dosage (PY) | 23.47 ± 22.77 | 19.45 ± 20.43 | 26.44 ± 26.53 | 25.15 ± 21.76 | - | - | 0.563 |  | - | - | - | - |
| - Past | 90 (26.5%) | 30 (30.0%) | 31 (25.8%) | 29 (24.2%) | 65 (40.9%) | 56 (54.1%) | 0.565 |  | **1.86**  (1.08 – 3.20) | **0.024** | **2.72**  (1.52 – 4.85) | **0.001** |
| - Dosage (PY) | 17.97 ± 20.19 | 16.74 ± 14.62 | 25.50 ± 20.30 | 15.28 ± 24.78 | **-** | **-** | **0.022** |  | - | - | - | - |
| Alcohol - Yes | 147 (43.2%) | 44 (44.0%) | 34 (28.3%) | 69 (57.7%) | 93 (58.5%) | 70 (64.2%) | **<0.001** |  | 1.04  (0.64 – 1.68) | 0.868 | 1.33  (0.78 – 2.26) | 0.299 |
| - Dosage (g/day) | 16.87 ± 15.90 | 19.24 ± 11.49 | 20.51 ± 20.92 | 13.57 ± 15.01 | **-** | **-** | **<0.001** |  | - | - | - | - |
| Coffee - Yes | 260 (76.7%) | 86 (86.0%) | 69 (57.5%) | 105 (88.2%) | 140 (88.1%) | 84 (77.1%) | **<0.001** |  | 0.98  (0.47 – 2.05) | 0.962 | **0.45**  (0.22 – 0.92) | **0.028** |
| - Dosage (cups) | 2.02 ± 1.48 | 1.14 ± 0.58 | 1.56 ± 0.74 | 3.07 ± 1.72 | 2.46 ± 1.21 | 2.69 ± 1.35 | **<0.001** |  | **-0.61**  (-0.99 – -0.24) | **0.001** | *-0.38*  *(-0.78 – 0.02)* | *0.060* |
| Other caffeine  - Yes | 71 (21.3%) | 8 (8.0%) | 22 (19.0%) | 41 (34.7%) | 60 (37.7%) | 34 (31.2%) | **<0.001** |  | 1.14  (0.69 – 1.87) | 0.609 | 0.85  (0.49 – 1.48) | 0.570 |
| - Dosage (cups) | 1.72 ± 1.30 | 1.00 ± 0.00 | 1.34 ± 0.75 | 2.04 ± 1.54 | **-** | **-** | **<0.001** |  | - | - | - | - |
| **Medications*** |  | | | | | | | | | | | |
| B-blockers | 48 (14.1%) | 18 (18.0%) | 5 (4.2%) | 25 (20.8%) | 57 (35.8%) | 22 (20.2%) | **<0.001** |  | **2.13**  (1.23 – 3.67) | **0.007** | 0.96  (0.51 – 1.83) | 0.903 |
| Antidepressants | 15 (4.4%) | 5 (5.0%) | 8 (6.7%) | 2 (1.7%) | 29 (18.2%) | 7 (6.4%) | 0.159 |  | **13.16**  (3.07 – 56.36) | **0.001** | 4.05  (0.82 – 19.93) | 0.085 |
| Benzodiazepines | 11 (3.2%) | 5 (5.0%) | 5 (4.2%) | 1 (0.8%) | 3 (1.9%) | 0 (0.0%) | 0.170 |  | 2.29  (0.24 – 22.28) | 0.476 | - | - |
| Other sleep drugs | 4 (1.2%) | 2 (2.0%) | 2 (1.7%) | 0 (0.0%) | 5 (3.1%) | 2 (1.8%) | 0.323 |  | - | - | - | - |

**Abbreviations:** Total Sibs = total cohort of siblings of PD patients; Italian Sibs = siblings coming from Azienda Unità Sanitaria Locale di Bologna – Istituto delle Scienze Neurologiche di Bologna (Italy); Spanish Sibs = siblings coming from Servicio Andaluz de Salud (Spain); German Sibs = siblings coming from Universitätsmedizin Göttingen (Germany); dnPDs = de novo PD patients from DeNoPa cohort; CTRs = controls from DeNoPa cohort; Adj. p = p adjusted for covariates; OR = odds ratio; CI = 95% confidence interval; β coef. = coefficient of regression from linear regression; PDSibs = sibling affected with Parkinson’s Disease; PY: pack/year; cup = equivalent of 125ml of liquid.

Continuous variables are expressed in mean ± standard deviation, discrete in number (%). Adjustments have been made for age, sex, education, smoking and coffee intake. Statistically significant coefficients and p-values are reported in **bold**. *These variables have not been adjusted.

**Supplementary Table 2.** Blood tests results of siblings and DeNoPa cohorts.

|  | **PROPAG-AGEING Siblings’ Cohort** | | | **DeNoPa Cohort** | | **Siblings’ Comparisons** | **Comparisons with DeNoPa** | | | |
| --- | --- | --- | --- | --- | --- | --- | --- | --- | --- | --- |
|  | **Sibs** | **Italian Sibs** | **German Sibs** | **dnPDs** | **CTRs** |  | **dnPDs vs. German Sibs** | | **CTRs vs. German Sibs** | |
| **Blood Biomarkers** |  | | | | | **Adj. p** | **β coef. (CI)** | **Adj. p** | **βcoef. (CI)** | **Adj. p** |
| WBC (x10^9^/L) | n=208 | n=100 | n=108 | n=159 | n=108 | **0.004** | 0.19  (-0.61 – 0.99) | 0.642 | -0.52  (-1.37 – 0.33) | 0.233 |
|  | 6.20 ± 1.67 | 6.42 ± 1.41 | 6.00 ± 1.86 | 6.36 ± 3.80 | 5.65 ± 1.45 |  |  |  |  |  |
| RBC (x10^12^/L) | n=208 | n=100 | n=108 | n=159 | n=108 | 0.663 | **-0.14**  (-0.25 – -0.03) | **0.015** | -0.10  (-0.22 – 0.02) | 0.091 |
|  | 4.86 ± 0.53 | 4.88 ± 0.61 | 4.84 ± 0.44 | 4.77 ± 0.48 | 4.81 ± 0.34 |  |  |  |  |  |
| Hemoglobin (g/dL) | n=208 | n=100 | n=108 | n=159 | n=107 | 0.607 | **-0.48**  (-0.79 – -0.17) | **0.003** | **-0.50**  (-0.83 – -0.16) | **0.004** |
|  | 14.63 ± 1.20 | 14.61 ± 1.23 | 14.65 ± 1.18 | 14.45 ± 1.42 | 14.51 ± 1.25 |  |  |  |  |  |
| MCV (fL) | n=208 | n=100 | n=108 | n=159 | n=108 | **0.001** | **-2.58**  (-3.93 – -1.23) | **<0.001** | **-2.71**  (-4.15 – -1.27) | **<0.001** |
|  | 88 ± 8 | 87 ± 9 | 90 ± 7 | 87± 4 | 87 ± 3 |  |  |  |  |  |
| Platelets (x10^9^/L) | n=208 | n=100 | n=108 | n=159 | n=108 | 0.148 | **-17.52**  (-34.87 – -0.17) | **0.048** | -11.34  (-29.84 – 7.16) | 0.229 |
|  | 249 ± 55 | 243 ± 54 | 254 ± 56 | 227 ± 70 | 234 ± 55 |  |  |  |  |  |
| Glucose (mmol/L) | n=187 | n=100 | n=87 | n=159 | n=108 | **<0.001** | **-0.73**  (-1.15 – -0.31) | **0.001** | **-0.86**  (-1.31 – -0.41) | **<0.001** |
|  | 5.51 ± 0.97 | 5.22 ± 0.84 | 5.83 ± 1.00 | 5.30 ± 1.07 | 5.10 ± 1.95 |  |  |  |  |  |
| Uric Acid (μmol/L) * | n=187 | n=100 | n=87 | n=159 | n=109 | 0.103 | **-28.99**  (-52.17 – -5.81) | **0.014** | -9.78  (-34.36 – 14.80) | 0.434 |
|  | 315.23 ± 88.05 | 305.44 ± 87.87 | 326.48 ± 87.40 | 320.03 ± 87.31 | 333.36 ± 83.94 |  |  |  |  |  |
| Creatinine (mg/dL) | n=186 | n=99 | n=87 | n=159 | n=109 | 0.155 | 0.04  (-0.01 – 0.09) | 0.093 | 0.01  (-0.04 – 0.06) | 0.686 |
|  | 0.80 ± 0.20 | 0.77 ± 0.20 | 0.82 ± 0.19 | 0.93 ± 0.20 | 0.89 ± 0.17 |  |  |  |  |  |
| Total Cholesterol (mg/dL) * | n=186 | n=99 | n=87 | n=159 | n=109 | 0.249 | -7.04  (-19.63 – 5.55) | 0.272 | **13.72**  (0.37 – 27.07) | **0.044** |
|  | 221.61 ± 42.15 | 218.26 ± 38.82 | 225.43 ± 45.72 | 212 ± 40 | 233 ± 40 |  |  |  |  |  |

**Abbreviations:** group acronyms are the same as explained in Table 1; Adj. p = p adjusted for covariates; β coef. = coefficient of regression from linear regression; CI = 95% confidence interval; WBC = White Blood Cells; RBC = Red Blood Cells; MCV = Mean Cell Volume.

Continuous variables are expressed in mean ± standard deviation. Adjustments have been made for age, sex, education, smoking and coffee intake. Statistically significant coefficients and p-values are reported in **bold**. *Additional adjustment for specific medications intake was performed.

**Supplementary Table 3.** Macrostructure sleep data of siblings and DeNoPa cohorts.

|  | **PROPAG-AGEING Siblings’ Cohort** | | | **DeNoPa Cohort** | | **Siblings’ Comparisons** | **Comparisons with DeNoPa** | | | |
| --- | --- | --- | --- | --- | --- | --- | --- | --- | --- | --- |
|  | **Sibs**  ***(n=147)*** | **Italian Sibs**  **(*n=98)*** | **German Sibs**  **(*n=49)*** | **dnPDs**  **(*n=159)*** | **CTRs**  **(*n=109)*** |  | **dnPDs vs.  German Sibs** | | **CTRs vs.  German Sibs** | |
| **Sleep - vPSG** |  | | | | | **Adj. p** | **βcoef. (CI)** | **Adj. p** | **βcoef. (CI)** | **Adj. p** |
| Total Sleep Time (min) | 342.72 ± 78.86 | 361.13 ± 74.38 | 305.91 ± 75.23 | **-** | **-** | **<0.001** | **-** | **-** | **-** | **-** |
| Sleep efficiency (%) | 74.47 ± 18.48 | 78.06 ± 18.56 | 67.31 ± 16.26 | 73.66 ± 10.63 | 74.34 ± 10.57 | **<0.001** | **8.64**  (4.60 – 12.68) | **<0.001** | **8.85**  (4.75 – 12.95) | **<0.001** |
| WASO (min) | 105.44 ± 59.82 | 100.19 ± 58.87 | 115.95 ± 61.50 | - | - | *0.072* | **-** | **-** | **-** | **-** |
| Sleep latency (min) | 16.79 ± 23.97 | 12.05 ± 19.42 | 26.28 ± 29.11 | 23.86 ± 19.05 | 22.95 ± 16.84 | **0.002** | -5.58  (-12.8 – 1.65) | 0.130 | -5.51  (-12.85 – 1.82) | 0.140 |
| REM latency (min) | 102.41 ± 63.72 | 93.25 ± 60.86 | 120.72 ± 63.72 | 107.27 ± 72.15 | 84.29 ± 47.18 | **0.003** | *-20.20*  (-42.33 – 1.93) | *0.074* | **-37.92**  (-60.38 – -15.47) | **0.001** |
| Stage 1 (%) | 17.90 ± 13.09 | 11.97 ± 7.02 | 29.77 ± 14.35 | 24.32 ± 10.68 | 27.41 ± 12.10 | **<0.001** | **-6.00**  (-10.08 – -1.93) | **0.004** | -2.67  (-6.81 – 1.46) | 0.204 |
| Stage 2 (%) | 42.74 ± 10.27 | 41.11 ± 8.91 | 46.02 ± 12.00 | 48.28 ± 10.50 | 47.47 ± 11.15 | **0.011** | *3.80*  (-0.23 – 7.83) | *0.065* | 2.14  (-1.95 – 6.23) | 0.304 |
| Stage 3 (%) | 19.98 ± 12.48 | 26.72 ± 8.58 | 6.50 ± 6.83 | 7.87 ± 8.48 | 7.41 ± 6.88 | **<0.001** | *2.26*  (-0.22 – 4.73) | *0.074* | 1.19  (-1.32 – 3.70) | 0.352 |
| Stage REM (%) | 19.83 ± 9.06 | 20.47 ± 7.69 | 18.56 ± 11.31 | 18.52 ± 6.75 | 17.70 ± 6.08 | 0.202 | -0.31  (-3.03 – 2.42) | 0.825 | -0.95  (-3.72 – 1.81) | 0.497 |
| PLM index - all | 19.58 ± 24.17 | 14.99 ± 18.33 | 28.29 ± 30.88 | 36.15 ± 38.20 | 33.06 ± 32.82 | **0.003** | 6.84  (-5.35 – 19.03) | 0.270 | 4.22  (-8.13 – 16.58) | 0.502 |
| - wake | - | - | 24.89 ± 37.54 | 37.10 ± 45.21 | 27.72 ± 33.75 | **-** | 10-16  (-3.59 – 23.91) | 0.147 | 1.31  (-12.65 – 15.26) | 0.854 |
| - sleep | - | - | 41.52 ± 39.20 | 29.56 ± 30.16 | 39.38 ± 38.38 | **-** | *-11.95*  (-24.44 – 0.53) | *0.061* | -1.92  (-14.57 – 10.74) | 0.766 |
| AHI | - | - | 5.71 ± 7.47 | 4.31 ± 7.69 | 3.11 ± 5.55 | **-** | -2.37  (-5.09 – 0.36) | 0.088 | **-3.16**  (-5.94 - -0.39) | **0.026** |
| SatO_2_ Min (%) | - | - | 86.54 ± 6.13 | 86.39 ± 7.36 | 86.43 ± 7.02 | **-** | 1.36  (-1.16 – 3.89) | 0.288 | 0.90  (-1.66 – 3.46) | 0.490 |

**Abbreviations:** group acronyms are the same as explained in Table 1; Adj. p = p adjusted for covariates; β coef. = coefficient of regression from linear regression; CI = 95% confidence interval; vPSG = video-polysomnography; min = minutes; WASO = Wake After Sleep Onset; PLM = Periodic Limb Movement; AHI = Apnea Hypopnea Index; Sat0_2_ Min = minimum value of oxygen saturation during sleep.

Continuous variables are expressed in mean ± standard deviation. Adjustments have been made for age, sex, education, smoking, coffee and sleep specific medications intake. Statistically significant coefficients and p-values are reported in **bold**.

**Supplementary Figure 1.** Association of prodromal PD markers in Siblings and Controls from DeNoPa cohort.

*Part A.* Association of the different prodromal PD markers in siblings (the only prodromal PD sibling, presenting RBD, constipation and slight motor symptoms is shown separately).
*The figure is a graphical representation of PD markers’ distribution rendered with Displayr | Analysis and Reporting Software for Survey Data (*[*https://www.displayr.com/*](https://www.displayr.com/)*), intersected areas represent subjects presenting with more than one marker.*

**
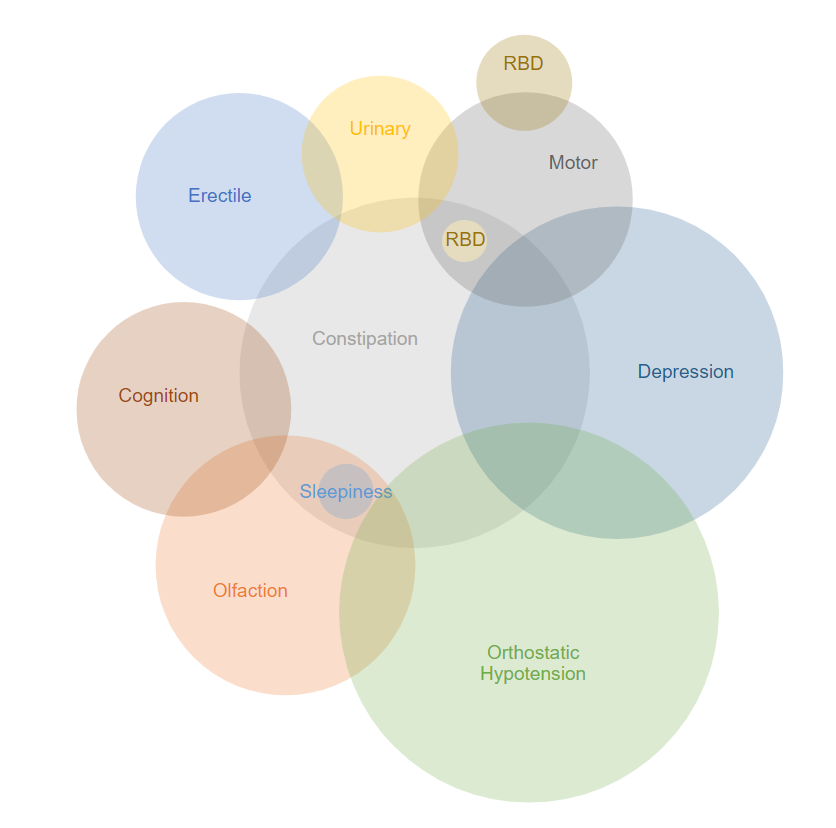
**

*Part B.* Association of the different prodromal PD markers in German Sibs (i) and CTRs (ii).
*The figure is a graphical representation of PD markers’ distribution rendered with Displayr | Analysis and Reporting Software for Survey Data (*[*https://www.displayr.com/*](https://www.displayr.com/)*), intersected areas represent subjects presenting with more than one marker.*

**i
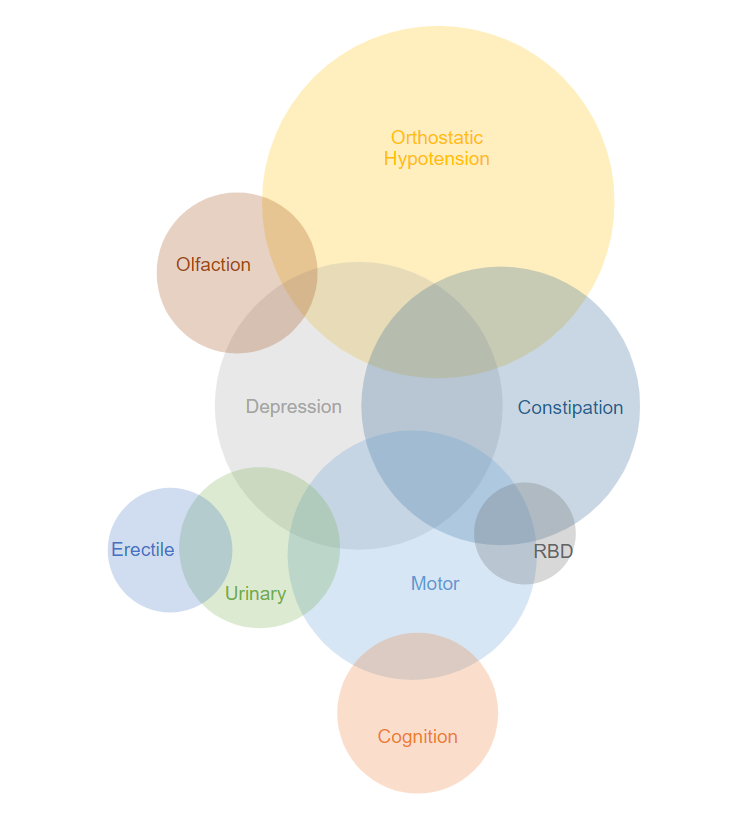
 ii
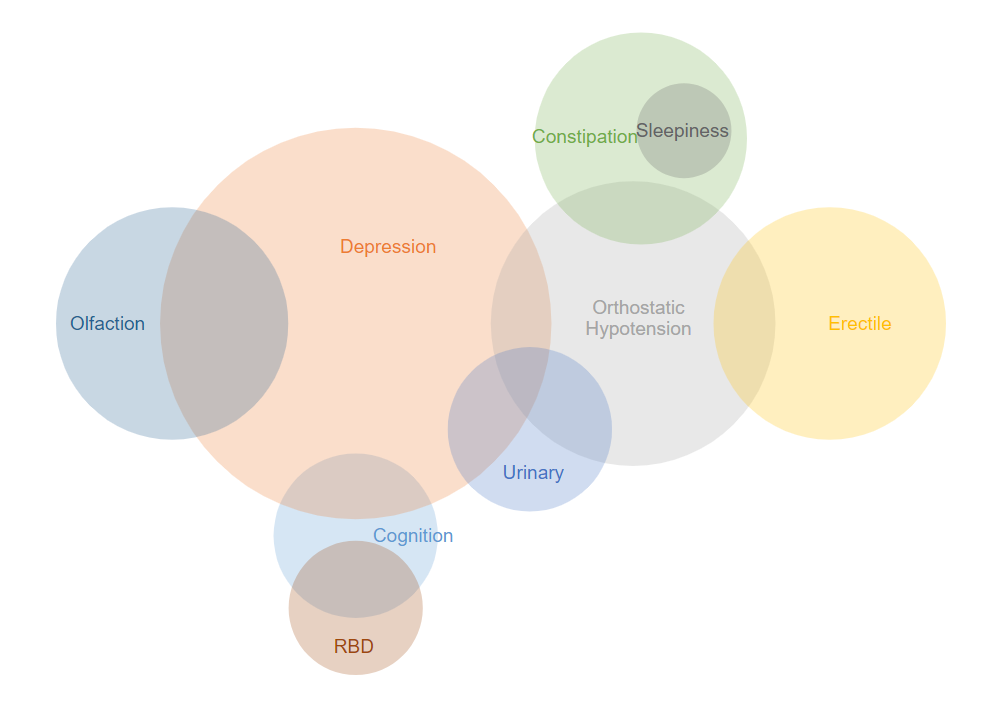
**
