# Supplementary material for: Heterogeneity of prodromal Parkinson symptoms in siblings of Parkinson disease patients
Source: NPJ Parkinsons Dis. 2021 Sep 7;7:78. doi: 10.1038/s41531-021-00219-1 (PMC8423761; doi:10.1038/s41531-021-00219-1)
Supplement: Supplementary file 2 — REPORTING SUMMARY [file 41531_2021_219_MOESM2_ESM.pdf]

nature research

Corresponding author(s): Federica Provini

Last updated by author(s): May 3, 2021

Reporting Summary

Nature Research wishes to improve the reproducibility of the work that we publish. This form provides structure for consistency and transparency in reporting. For further information on Nature Research policies, see our Editorial Policies and the Editorial Policy Checklist.

Statistics

For all statistical analyses, confirm that the following items are present in the figure legend, table legend, main text, or Methods section.

n/a	Confirmed	
☐	☒	The exact sample size (*n*) for each experimental group/condition, given as a discrete number and unit of measurement
☐	☒	A statement on whether measurements were taken from distinct samples or whether the same sample was measured repeatedly
☐	☒	The statistical test(s) used AND whether they are one- or two-sided<br>*Only common tests should be described solely by name; describe more complex techniques in the Methods section.*
☐	☒	A description of all covariates tested
☐	☒	A description of any assumptions or corrections, such as tests of normality and adjustment for multiple comparisons
☐	☒	A full description of the statistical parameters including central tendency (e.g. means) or other basic estimates (e.g. regression coefficient) AND variation (e.g. standard deviation) or associated estimates of uncertainty (e.g. confidence intervals)
☐	☒	For null hypothesis testing, the test statistic (e.g. F, t, r) with confidence intervals, effect sizes, degrees of freedom and P value noted<br>*Give P values as exact values whenever suitable.*
☒	☐	For Bayesian analysis, information on the choice of priors and Markov chain Monte Carlo settings
☒	☐	For hierarchical and complex designs, identification of the appropriate level for tests and full reporting of outcomes
☒	☐	Estimates of effect sizes (e.g. Cohen's *d*, Pearson's *r*), indicating how they were calculated

Our web collection on statistics for biologists contains articles on many of the points above.

Software and code

Policy information about availability of computer code

Data collection	Online shared database accessible upon autentication.
Data analysis	Statistical analysis was performed using SPSS Statistics version 21 (IBM, Armonk, NY, USA) and Stata SE version 14.2 (StataCorp LLC, Texas, USA).

For manuscripts utilizing custom algorithms or software that are central to the research but not yet described in published literature, software must be made available to editors and reviewers. We strongly encourage code deposition in a community repository (e.g. GitHub). See the Nature Research guidelines for submitting code & software for further information.

Data

Policy information about availability of data

All manuscripts must include a data availability statement. This statement should provide the following information, where applicable:

- Accession codes, unique identifiers, or web links for publicly available datasets
- A list of figures that have associated raw data
- A description of any restrictions on data availability

Anonymized data will be shared by request from any qualified investigator.

Field-specific reporting

Please select the one below that is the best fit for your research. If you are not sure, read the appropriate sections before making your selection.

☒ Life sciences ☐ Behavioural & social sciences ☐ Ecological, evolutionary & environmental sciences

For a reference copy of the document with all sections, see nature.com/documents/nr-reporting-summary-flat.pdf

Life sciences study design

All studies must disclose on these points even when the disclosure is negative.

Sample size	Sample size calculation was based on the recruiting centres recruiting force, in order to ensure a smooth enrolment.
Data exclusions	Participants had to be over 18 years of age and could not have any active known/treated condition of the central nervous system (e.g., Alzheimer’s disease, vascular encephalopathies, multiple sclerosis) including Parkinson disease. All the possible participants with these conditions were excluded prior to enrollment.
Replication	Validated scales, questionnaires and scoring were used in order du ensure reproducibility.
Randomization	Not relevant for sthe study as it is a cross-sectional non-pharmacological study.
Blinding	Not relevant for sthe study as it is a cross-sectional non-pharmacological study.

Reporting for specific materials, systems and methods

We require information from authors about some types of materials, experimental systems and methods used in many studies. Here, indicate whether each material, system or method listed is relevant to your study. If you are not sure if a list item applies to your research, read the appropriate section before selecting a response.

Materials & experimental systems

n/a	Involved in the study	
☒	☐	Antibodies
☒	☐	Eukaryotic cell lines
☒	☐	Palaeontology and archaeology
☒	☐	Animals and other organisms
☐	☒	Human research participants
☒	☐	Clinical data
☒	☐	Dual use research of concern

Methods

n/a	Involved in the study	
☒	☐	ChIP-seq
☒	☐	Flow cytometry
☒	☐	MRI-based neuroimaging

Human research participants

Policy information about studies involving human research participants

Population characteristics	A total of 340 siblings (n=141; 41.5% males) were included in the study from the three recruiting centers, their mean age was 62.13 ± 10.72; SAS siblings (Spanish Sibs) were the youngest aged 57.77 ± 11.17 years (p<0.001). Alcohol and coffee consumption were more frequent among German Sibs (57.7% and 88.2% respectively), while Spanish Sibs had the highest per capita alcohol daily dose (20.51 ± 20.92 g/day; p<0.001). Regarding comorbidities, hypothyroidism was more prevalent among German Sibs (19.2%; p<0.001).
Recruitment	We report the description of the PPG siblings cohort (Sibs) multicentrically recruited over 29 months between September 2016 and January 2019 by three PPG partners: Azienda Unità Sanitaria Locale di Bologna – Istituto delle Scienze Neurologiche di Bologna (ISNB, Italy), Servicio Andaluz de Salud (SAS, Spain) and Paracelsus-Elena Hospital, Kassel as part of Universitätsmedizin Göttingen (UMG-GOE, Germany). To account for population-specific effects we subsequently compared sibling data from UMG-GOE (German-Sibs) with the baseline data of de novo PD patients (dnPDs) and controls (CTRs) from the DeNoPa cohort from the same center.
Ethics oversight	We conducted the study according to the Declaration of Helsinki and all participants provided informed written consent. The study was approved by the local ethics committees of each recruiting PPG partner (UMG-GOE ethics committee no. of approval 19/5/16 of August 2016, ISNB ethics committee no. of approval 16018 of May 2016, SAS ethical committee no. of approval 2014/PI173 of September 2016).

Note that full information on the approval of the study protocol must also be provided in the manuscript.
